# Soluble Interleukin-2 Receptor Level as a Marker of Hemophagocytic Lymphohistiocytosis in Children With Severe Dengue

**DOI:** 10.3389/fped.2021.721857

**Published:** 2021-10-27

**Authors:** Dhirendra Singh, Veena Raghunathan, Maninder Dhaliwal, Neha Rastogi, Ritu Chadha, Satya Prakash Yadav

**Affiliations:** Medanta the Medicity Hospital, Gurgaon, India

**Keywords:** dengue, severe dengue, pediatrics, hemophagocytic lymphohistiocytosis, soluble interleukin-2 receptor (IL2R)

## Abstract

Dengue induced-hemophagocytic lymphohistiocytosis (HLH) is increasingly recognized as an important cause of secondary HLH. Early identification of dengue HLH and directed therapy for HLH may help to alter the outcomes in critically ill patients. Soluble interleukin-2 receptor (IL2R) is a useful inflammatory marker and is seen to correlate with HLH disease activity. There is scarcity of data on IL2R in pediatric dengue patients with HLH. All patients (age < 18 years) with severe dengue confirmed by positive dengue IgM ELISA admitted to PICU were retrospectively enrolled. Patientswere screened for presence of HLH according to HLH 2004 criteria. Hemogram, ferritin, fibrinogen, liver, and renal function tests were noted. Patients who met four or more HLH criteria were treated with steroids and IL2R levels were sent to confirm the diagnosis of HLH. Out of 15 patients, nine patients met the criteria of HLH. IL2R levels were high in all HLH patients (mean 51,711, range 18,000–98,715 pg/mL). Mean ferritin levels were high in the HLH group as compared to non-HLH group (mean ferritin 34,593 vs. 3,206 ng/mL; *p*-value 0.004). Liver dysfunction was notably higher in the HLH group compared to non-HLH group (mean alanine aminotransferase 6,621 U/L vs. 165.6 U/L; *p*-value 0.04, mean aspartate aminotransferase 2,145 U/L vs. 104.2 U/L; 0.04, bilirubin level 4.2 mg/dL vs. 0.7 mg/dL; *p*-value 0.03). Four patients in the HLH group had acute kidney injury (AKI) and two required renal replacement therapy in the form of sustained low efficiency dialysis (SLED). Requirement for invasive ventilation was exclusively seen in HLH group and three patients developed ARDS. Two patients each in HLH and non-HLH group had shock requiring vasoactive therapy in addition to fluids. Mean days of ICU and hospital stay were higher in HLH group vs. non-HLH group but not statistically significant (6.4 vs. 4.4; *p*-value 0.32 and 8.44 vs. 5.6; *p*-value 0.18 days, respectively). All children in HLH group received steroids as per HLH protocol. In the HLH group, seven survived while two died. In the non-HLH group, all five patients survived. We concluded that IL2R levels are high in dengue HLH and useful for definitive diagnosis. Early recognition of this condition in severe dengue and prompt steroid therapy improves chances of better outcome.

## Introduction

### Background

Dengue is a viral infection which can lead to severe illness causing multiorgan dysfunction and mortality. It has no specific treatment and management is essentially supportive. Dengue-induced hemophagocytic lymphohistiocytosis (HLH) is increasingly recognized as an important cause of secondary HLH (1). HLH leads to overactivation of the immune system and cytokine storm, thus precipitating multiorgan failure. Recognition of HLH can be a diagnostic challenge in the background of dengue, as its clinical features can mimic sepsis and liver failure (2). However, early identification of dengue HLH is important as directed therapy for HLH may help to alter the outcomes in critically ill patients. Soluble interleukin-2 receptor (IL2R), also known as soluble CD 25, is a useful inflammatory marker and has been shown to correlate with HLH disease activity (3). The interleukin-2 receptor is a transmembrane protein which is upregulated on activated T cells, hence high IL2R levels can be found in conditions associated with T cell activation, including HLH, autoimmune lymphoproliferative syndrome, etc. (4). Interleukin-2 induces lymphokine-activated killer cells and thromboxane-A2 which may alter endothelial permeability and plasma leakage. IL2Ris elevated in both primary and secondary forms of HLH and hence is included in the diagnostic criteria of HLH (5). To our knowledge, this is the first study which reports IL2R levels in pediatric dengue HLH.

### Aim

To study the IL2R levels in pediatric patients (<18 years) with severe dengue with HLH.

### Secondary Aim

To compare the clinical and laboratory characteristics of patients with severe dengue with and without HLH.

## Methods

All pediatric patients with severe dengue confirmed by positive dengue IgM ELISA or NS1 antigen admitted to our PICU were retrospectively enrolled. Severe dengue was defined by the presence of or plasma leakage causing shock/respiratory distress or severe bleeding or severe organ impairment according to WHO 2009 classification (6). These patients were screened for the presence of HLH according to the HLH 2004 criteria (7). Patients in whom five or greater criteria were met were diagnosed with HLH. All patients with severe dengue who met four or more HLH criteria were treated with steroids and IL2R levels were sent in them to confirm HLH. The rest of the patients with severe dengue (with three or less criteria satisfied) were managed as per WHO standard protocol without steroids (6). Their clinical and laboratory parameters were compared with the severe dengue HLH patient group.

Hemogram, ferritin, fibrinogen, serum triglycerides, liver, and renal function tests were noted in all patients. Ultrasound abdomen was done to look for splenomegaly. Bone marrow was planned only if needed for the diagnosis of HLH in hemodynamically stable children. IL2R was measured in all patients with suspected HLH (patients who satisfied at least 4 HLH criteria). It was measured by either eBiosecience or Daclone Human soluble IL-2 ELISA kit. These are both enzyme-linked immunosorbent assays for quantitative detection of IL2R level in human serum. Clinical details including pediatric risk of mortality (PRISM) score, presence of shock requiring vasoactive therapy, need for invasive and non-invasive ventilation, comorbidities, and outcomes were noted in all patients.

### Ethics

The Institutional Ethics Committee waived the need of ethic approval and the need to obtain consent for collection, analysis, and publication of retrospectively obtained and anonymized data for this non-interventional study.

## Results

Fifteen patients with severe dengue were admitted in PICU between December 2019 and December 2020. Table 1 shows the demographic, anthropometric, and laboratory characteristics of patient with severe dengue with and without HLH.

**Table 1:** Demographic, anthropometric, and laboratory characteristics of patient with severe dengue with and without HLH.

	**Severe dengue with HLH (*N* = 9)**	**Severe dengue without HLH (*N* = 5)**	***p*-value**
Mean age (years) ± SD	10.44 ± 5.22	13.4 ± 4.56	0.29
Male, n%	8 (88.8%)	4 (80%)	1
Mean BMI (kg/m^2^) ± SD	25.11 ± 7.64	22.36 ± 5.61	0.46
Mean BMI (Z-score) ± SD	1.72 ± 1.51	0.96 ± 1.26	0.33
Mean platelet count (per mm^3^) ± SD	49,111 ± 29,135.22	51,800 ± 58,104.45	0.93
SGOT (U/L) ± SD	6,621.55 ± 8,150.75	165.6 ± 132.73	0.04
Worst mean total bilirubin (mg/dl) ± SD	4.21 ± 4.01	0.7 ± 0.32	0.03
Mean ferritin (ng/mL) ± SD	34,593.22 ± 23,950.43	3,206.46 ± 3,195.664	0.004
Mean SGPT (U/L) ± SD	1,979.25 ± 2,416.24	104.2 ± 88.82	0.04
Mean IL2R (pg/mL) ± SD	57,111 ± 23,910	Not done	
Mean PRISM 3 at 24 h ± SD	12.37 ± 13.17	4.8 ± 6.14	0.19
Mean PRISM 3 at 12 h ± SD	11 ± 6	6 ± 4.69	0.11
Mean LDH at admission (U/L) ± SD	7929.78 ± 10,811.8	542.4 ± 337.1184	0.07
Worst mean albumin (gm/dL) ± SD	2.49 ± 0.73	3.52 ± 1.34	0.17
Worst mean urea (mg/dL) ± SD	83.22 ± 83.93	20.8 ± 4.76	0.06
Worst mean creatinine (mg/dL) ± SD	1.53 ± 1.63	0.7 ± 0.21	0.17
Mean ICU stay (days) ± SD	6.44 ± 4.61	4.4 ± 2.79	0.32
Mean hospital stay (days) ± SD	8.44 ± 5.25	5.6 ± 2.07	0.18
Need for ventilation, n%	6 (66%)	1 (20%)	0.26
Mean Invasive ventilation days ± SD	2.88 ± 4.34	0 ± 0	0.08
Mean HFNC days ± SD	1.89 ± 2.09	0.8 ± 1.79	0.32
Vasoactive therapy besides fluids, *n* (%)	2 (22%)	0 (0%)	0.5
Outcome: number of patients who died *n* (%)	2 (22%)	0 (0%)	0.5

*ALT, alanine aminotransferase; AST, aspartate transaminase; HFNC, high flow nasal cannula*.

Out of 15 enrolled patients, nine met the criteria for HLH and IL2R levels were raised in all nine patients. IL2R levels could not be done in one additional patient with suspected HLH, who satisfied four out of eight criteria as he had a catastrophic course and expired within 12 h of admission. This patient was hence excluded from analysis. Among the nine patients, seven (77%) satisfied 5 HLH criteria and the remaining two satisfied 6 criteria. The remaining five patients with severe dengue satisfied only three or lesser HLH criteria and were hence classified into non-HLH group. Among the nine patients with confirmed HLH, all had low platelet counts (<1 lakh/mm^3^) and high ferritin levels >5,000 ng/mL except one (829 ng/mL). IL2R levels were high in all HLH patients (mean 51,711, range 18,000–98,715 pg/mL). Lactate dehydrogenase levels were high in all patients (range 638–34,191 U/L). Splenomegaly was present in all nine except one patient. Only one patient in the HLH group had evidence of hemophagocytosis on peripheral smear. Bone marrow examination could not be performed in any of the patients due to hemodynamic instability or severe respiratory distress. Four patients in the HLH group had AKI and 2 required renal replacement therapy in the form of sustained low efficiency dialysis (SLED). None of the patients in non-HLH group had AKI. Admission urea was higher in HLH group (mean urea 83.22 mg/dL) as compared to non-HLH group (mean urea −27 mg/dL) but did not reach a significant level (*p*-value 0.06 and 0.17, respectively).

Mean ferritin levels were 34,593 ng/mL in the HLH group vs. 3,206 ng/mL in the non-HLH group (*p*-value 0.004). Liver dysfunction was notably higher in the HLH group compared to non-HLH group (meanalanine aminotransferase 6,621 U/L vs. 165.6 U/L; *p*-value 0.04, mean aspartate aminotransferase 2,145 U/L vs. 104.2 U/L; 0.04, bilirubin level 4.2 mg/dL vs. 0.7 mg/dL; *p*-value 0.03).

Four patients in the HLH group required invasive ventilation and additional two patients required high concentration of oxygen delivered via high flow nasal cannulae. In the non-HLH group, one patient required high flow nasal cannulae oxygen delivery and none required invasive ventilation. Three patients in the HLH group developed ARDS. Two patients each in HLH and non-HLH group had shock requiring vasoactive therapy in addition to fluids. Mean days of ICU stay and hospital stay were higher in HLH group vs. non-HLH group but did not reach a statistical significant level (6.4 vs. 4.4; *p*-value 0.32 and 8.44 vs. 5.6; *p*-value 0.18 days, respectively).

All nine children in HLH group received steroids as per HLH protocol (4). In the HLH group, seven survived while two died. In the non-HLH group, all five patients survived.

## Discussion

Dengue infection is being increasingly recognized as a cause of infection-induced HLH, especially in the endemic areas, and has been reported to account for 26% of virus-associated HLH (8). Persistence of fever is unusual in dengue and its presence along with very high ferritin >10,000 ng/mL should be considered as an important clue towards underlying HLH (9, 10). Patients with dengue HLH who often get sick land up in the PICU in a very critical condition with evidence of organ dysfunction. Time is of utmost importance in such patients, as evaluation and formal diagnosis of HLH may consume time, leading to delay in initiation of HLH therapy.

The HLH 2004 criteria mandates that five out of eight criteria should be fulfilled for definitive diagnosis of HLH. While five of these criteria (fever, splenomegaly, cytopenias, hypertriglyceridemia/hypofibrinogenemia, and hyperferritinemia) can be easily obtained on routine clinical and laboratory assessment, they may not all be fulfilled always. Thus, a patient who falls short of any of these five criteria cannot be definitively diagnosed with HLH. This is often the case in a patient with severe dengue, when HLH is suspected, but cannot be proved as <5 criteria are satisfied. The sick condition of the patient makes it difficult to perform bone marrow examination for diagnosis. In such a scenario, steroid therapy may be delayed or withheld which can adversely affect the outcomes.

The remaining 3 HLH criteria which can be useful for diagnosis are natural killer cell function, IL2R, and genetic testing. Of these, genetic testing is an expensive and time-consuming process, hence, it is of limited practical value in the acute setting. Natural killer function testing requires radioactive reagents and specialized flow cytometry techniques. These are both specialized tests and not easily available. On the other hand, IL2R testing is advantageous, as it is simple, inexpensive, and requires commercially available assay and provides quick results. Since we had access to IL2R levels, definitive diagnosis of HLH in our patients became easier. In the HLH group, 77% of patients satisfied five out of eight criteria and would have fallen short of definitive diagnosis in its absence.

Extreme hyperferritenemia is traditionally considered as an important clue to HLH. In our case series too, patients in HLH group had higher mean ferritin levels compared to non-HLH group. In certain forms of secondary HLH, IL2R has been found to correlate with disease severity more consistently than ferritin (3). Its specific role in dengue requires further close scrutiny. Kumar et al. could not establish any significant association between IL-2 levels and severity of dengue (11). We found IL2R levels to be consistently high in dengue HLH and these patients had higher PRISM scores than the non-HLH group. IL2R may have a prognostic role as levels >10,000 U/mL have been associated with poor outcomes in HLH (12).

In patients with severe dengue, life-threatening organ dysfunction can occur. Distinguishing HLH from the myriad clinical presentations of severe dengue is, therefore, difficult; however, certain pointers exist. We noted that patients with HLH were sicker at presentation (higher PRISM scores) and had higher severity of liver, kidney dysfunction, and ARDS. The need for invasive ventilation was exclusively seen in HLH group. Liver involvement was particularly marked, with significantly higher bilirubin and transaminase levels. These findings resonate with other pediatric studies which have found hypoalbuminemia, elevated alanine aminotransferase, and severe organ dysfunction to be significantly higher in patients with severe dengue with HLH than those without HLH (13). Longer duration of fever, longer duration of hospitalization, and higher need for PICU admission have also been linked to the presence of underlying HLH in dengue (9). We also found that mortality exclusively occurred in the HLH group, thus, possibly suggesting that identification of HLH may play a prognostic role too.

Our study is small and retrospective in nature. Due to practical issues, we were not able to carry out genetic testing, natural killer function tests in our patients. While IL2R was high in all patients in HLH group, the numbers are small and the lack of comparative values makes it impossible to identify cut offs specific to dengue HLH in children. More studies are needed to understand dengue HLH better and define the true role of IL2R and other cytokine.

## Conclusion

HLH is rare but is a severe complication of severe dengue. Patients with severe dengue and HLH have high ferritin levels and can develop severe multiorgan dysfunction with poor outcomes. IL2R levels are high in dengue HLH and thus useful for definitive diagnosis of HLH. Since it is an easily implemented test with high diagnostic value, it should be incorporated in future studies. Early identification of HLH in severe dengue by sensitive markers is important to initiate specific therapy in the form of steroids, which are not otherwise used in dengue, to improve chances of a better outcome.

## Data Availability Statement

The raw data supporting the conclusions of this article will be made available by the authors, without undue reservation.

## Ethics Statement

The Institutional Ethics Committee waived the need of ethic approval and the need to obtain consent for collection, analysis and publication of retrospectively obtained and anonymize data for this non-interventional study.

## Author Contributions

DS: collected the data and manuscript writing. VR: conceived and designed the analysis. MD: performed the analysis and supervised the research. NR: developed the theoretical framework. RC: performed the IL2R test. SY: helped shape the research, analysis, and manuscript. All authors contributed to the article and approved the submitted version.

## Conflict of Interest

The authors declare that the research was conducted in the absence of any commercial or financial relationships that could be construed as a potential conflict of interest.

## Publisher's Note

All claims expressed in this article are solely those of the authors and do not necessarily represent those of their affiliated organizations, or those of the publisher, the editors and the reviewers. Any product that may be evaluated in this article, or claim that may be made by its manufacturer, is not guaranteed or endorsed by the publisher.
